# Supplementary material for: The association between ABO blood group and SARS-CoV-2 infection: A meta-analysis
Source: PLoS One. 2020 Sep 18;15(9):e0239508. doi: 10.1371/journal.pone.0239508 (PMC7500631; doi:10.1371/journal.pone.0239508)

**ABO polymorphism and SARS-CoV-2 infection - a meta-analysis**

**1.Eligibility Criteria**

The inclusion criteria were as follows:

**Population**: The unit of analysis of this review is studies published in health-related journals with original data or results.

**Interventions/exposures**: We included studies that describe the distribution of AB0 blood groups in SARS-CoV-2 positive.

**Comparator**: ABO blood group distribution among either the general healthy or COVID-19 negative population of the related geographic area.

**Outcome measures**: To be included, studies had to report or allow to calculate Odds Ratios (ORs) for the association of testing positive for SARS-CoV-2 and having a specific blood group (A, B, AB, or O).

**Study design**: Experimental studies (e.g. nonrandomized studies or cohort studies), observational studies, case series, and case studies.

**Publication type**: We included original research papers, including scientific meeting abstracts or research letters, if they contained sufficient information to fill the extraction forms.

**2.Search strategy**

(search date: July 16, 2020)

| **Search strategy** | **Output** |
| --- | --- |
| (COVID-19 OR SARS-CoV-2) AND (“blood group” OR “ABO”) | MEDLINE (n= 42) |
| ”COVID-19” OR “SARS-CoV-2” AND “blood group” OR “ABO” | LITCOVID (n=13) |
| **TOTAL** | **n=55** |

**3.Content**

**Table S1.** Quality of studies assessment: Newcastle Ottawa Scale

|  | **Li**  **1** | **Zietz**  **2** | **Göker**  **3** | **Wu**  **4** | **Ellinghaus**  **5a,5b** | **Leaf**  **6a,6b,6c,6d** | **Dzik**  **7** |
| --- | --- | --- | --- | --- | --- | --- | --- |
| **Selection** |  |  |  |  |  |  |  |
| 1) Is the case definition adequate?  a) yes, with independent validation *  b) yes, eg record linkage or based on self reports  c) no description | b | b | b | b | b | b | b |
| 2) Representativeness of the cases  a) consecutive or obviously representative series of cases *  b) potential for selection biases or not stated | b | b | b | b | b | b | b |
| 3) Selection of Controls  a) community controls *  b) hospital controls  c) no description | a | a | a | b | a | a | b |
| 4) Definition of Controls  a) no history of disease (endpoint) *  b) no description of source | a | a | a | a | a | a | a |
| **Comparability** |  |  |  |  |  |  |  |
| 1) Comparability of cases and controls on the basis of the design or analysis  a) study controls for_______ (Select the most important factor.) *  b) study controls for any additional factor * (This criteria could be modified to indicate specific control for a second important factor.) | a | a | a | a | a | a | a |
| **Exposure** |  |  |  |  |  |  |  |
| 1) Ascertainment of exposure  a) secure record (eg surgical records) *  b) structured interview where blind to case/control status *  c) interview not blinded to case/control status  d) written self report or medical record only  e) no description | a | a | a | a | a | a | a |
| 2) Same method of ascertainment for cases and controls  a) yes *  b) no | a | a | a | a | a | a | a |
| 3) Non-Response rate  a) same rate for both groups *  b) non respondents described  c) rate different and no designation | N/A | N/A | N/A | N/A | N/A | N/A | N/A |
| **TOTAL (OUT OF 8)** | **5** | **5** | **5** | **4** | **5** | **5** | **4** |
| **SCORE %** | 62.5 | 62.5 | 62.5 | 50.0 | 62.5 | 62.5 | 50.0 |

**4.Sensitivity analysis (leave-one-out approach)**

**Table S2**. Results from sensitivity analysis leave-one-out method: pooled OR and 95% confidence interval calculated omitting each study in turn

|  | A vs AB·B·0 | | B vs A·AB·0 | | AB vs A·B·0 | | 0 vs A·B·AB | |
| --- | --- | --- | --- | --- | --- | --- | --- | --- |
| Study omitted | pooled OR | 95%CI | pooled OR | 95%CI | pooled OR | 95%CI | pooled OR | 95%CI |
| 1a (Li) | 1·232 | 1·067 - 1·423 | 1·042 | 0·935 - 1·161 | 1·079 | 0·904 - 1·287 | 0·779 | 0·676 - 0·897 |
| 1b (Li) | 1·226 | 1·074 - 1·398 | 1·060 | 0·965 - 1·164 | 1·065 | 0·915 - 1·239 | 0·776 | 0·677 - 0·890 |
| 1c (Li) | 1·225 | 1·071 - 1·400 | 1·052 | 0·953 - 1·162 | 1·085 | 0·923 - 1·275 | 0·777 | 0·676 - 0·893 |
| 2 (Zietz) | 1·253 | 1·091 - 1·439 | 1·037 | 0·938 - 1·146 | 1·124 | 0·975 - 1·296 | 0·755 | 0·653 - 0·874 |
| 3 (Göker) | 1·185 | 1·054 - 1·332 | 1·068 | 0·979 - 1·166 | 1·110 | 0·956 - 1·288 | 0·786 | 0·687 - 0·900 |
| 4 (Wu) | 1·215 | 1·066 - 1·385 | 1·049 | 0·951 - 1·158 | 1·110 | 0·957 - 1·288 | 0·777 | 0·677 - 0·892 |
| 5a (Ellinghaus) | 1·207 | 1·061 - 1·372 | 1·078 | 0·987 - 1·176 | 1·067 | 0·911 - 1·250 | 0·779 | 0·676 - 0·897 |
| 5b (Ellinghaus) | 1·228 | 1·071 - 1·408 | 1·038 | 0·947 - 1·138 | 1·055 | 0·917 - 1·214 | 0·783 | 0·682 - 0·899 |
| 6a (Leaf) | 1·234 | 1·073 - 1·419 | 1·049 | 0·949 - 1·160 | 1·055 | 0·904 - 1·230 | 0·771 | 0·667 - 0·891 |
| 6b (Leaf) | 1·252 | 1·091 - 1·437 | 1·039 | 0·938 - 1·152 | 1·121 | 0·968 - 1·297 | 0·755 | 0·653 - 0·873 |
| 6c (Leaf) | 1·247 | 1·093 - 1·422 | 1·039 | 0·949 - 1·139 | 1·072 | 0·919 - 1·251 | 0·777 | 0·677 - 0·891 |
| 6d (Leaf) | 1·265 | 1·110 - 1·441 | 1·080 | 0·999 - 1·168 | 1·090 | 0·932 - 1·275 | 0·741 | 0·654 - 0·840 |
| 7 (Dzik) | 1·277 | 1·143 - 1·426 | 1·039 | 0·938 - 1·150 | 1·084 | 0·919 - 1·279 | 0·746 | 0·658 - 0·846 |
| None | 1·234 | 1·087 - 1·400 | 1·052 | 0·960 - 1·153 | 1·086 | 0·936 - 1·259 | 0·769 | 0·674 - 0·879 |

**5.Subgroup analysis**

**Figure S1.** Forest plot from random effects analysis: OR of being blood group A in SARS-CoV-2+ group versus control group, by type of Control population.

**
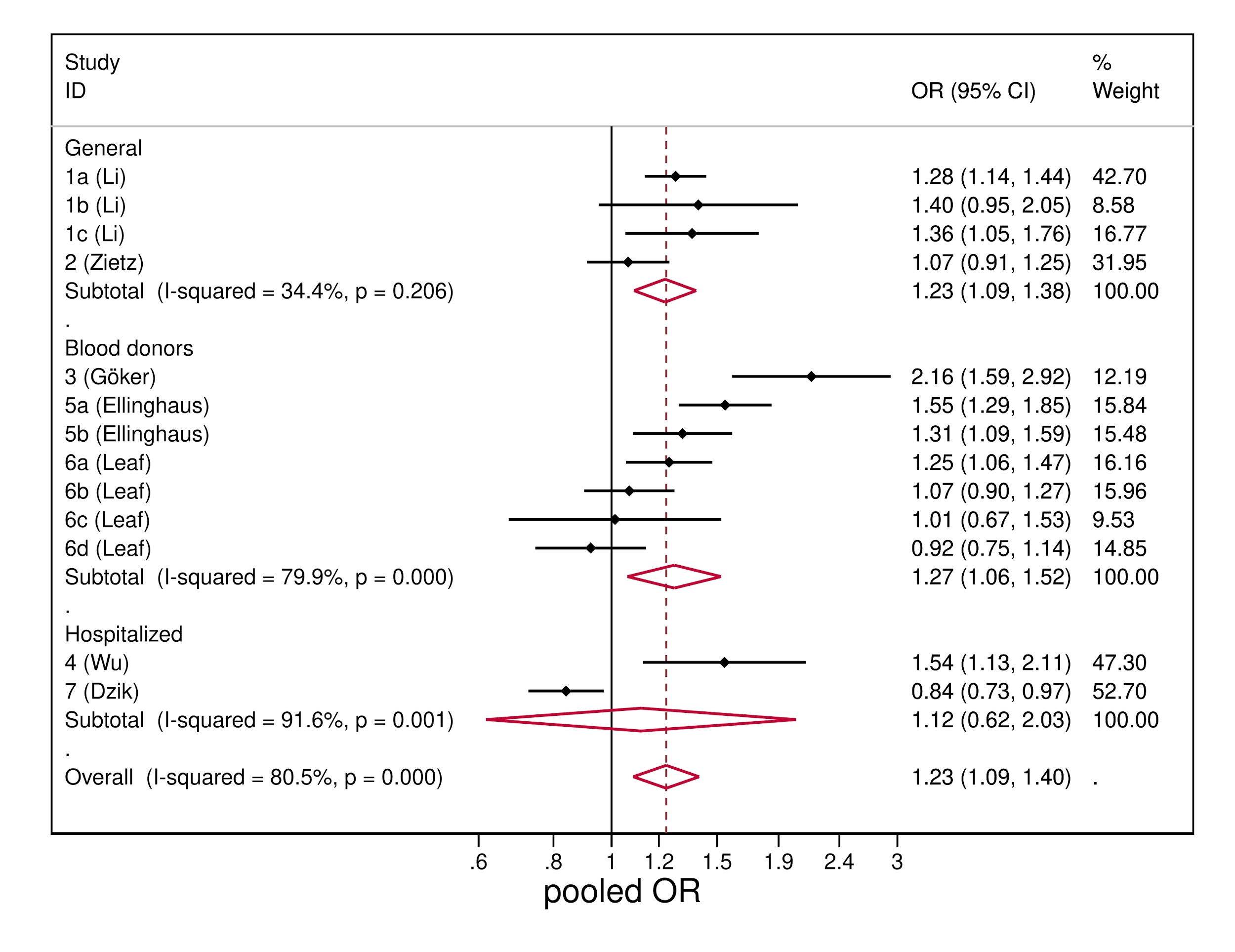
**

**Figure S2.** Forest plot from random effects analysis: OR of being blood group A in SARS-CoV-2+ group versus control group, by Country.


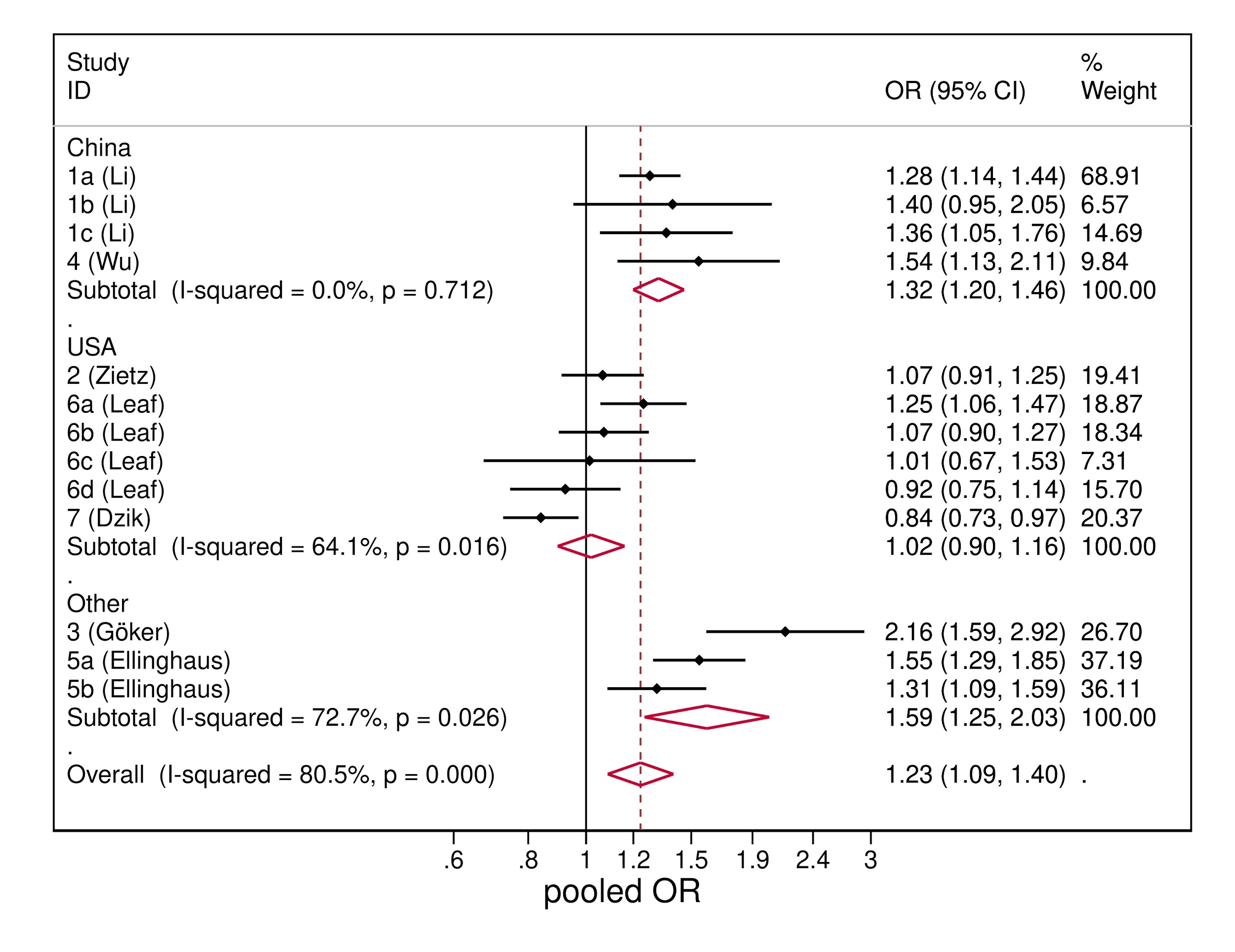


**Figure S3.** Forest plot from random effects analysis: OR of being blood group 0 in SARS-CoV-2+ group versus control group, by type of control population.


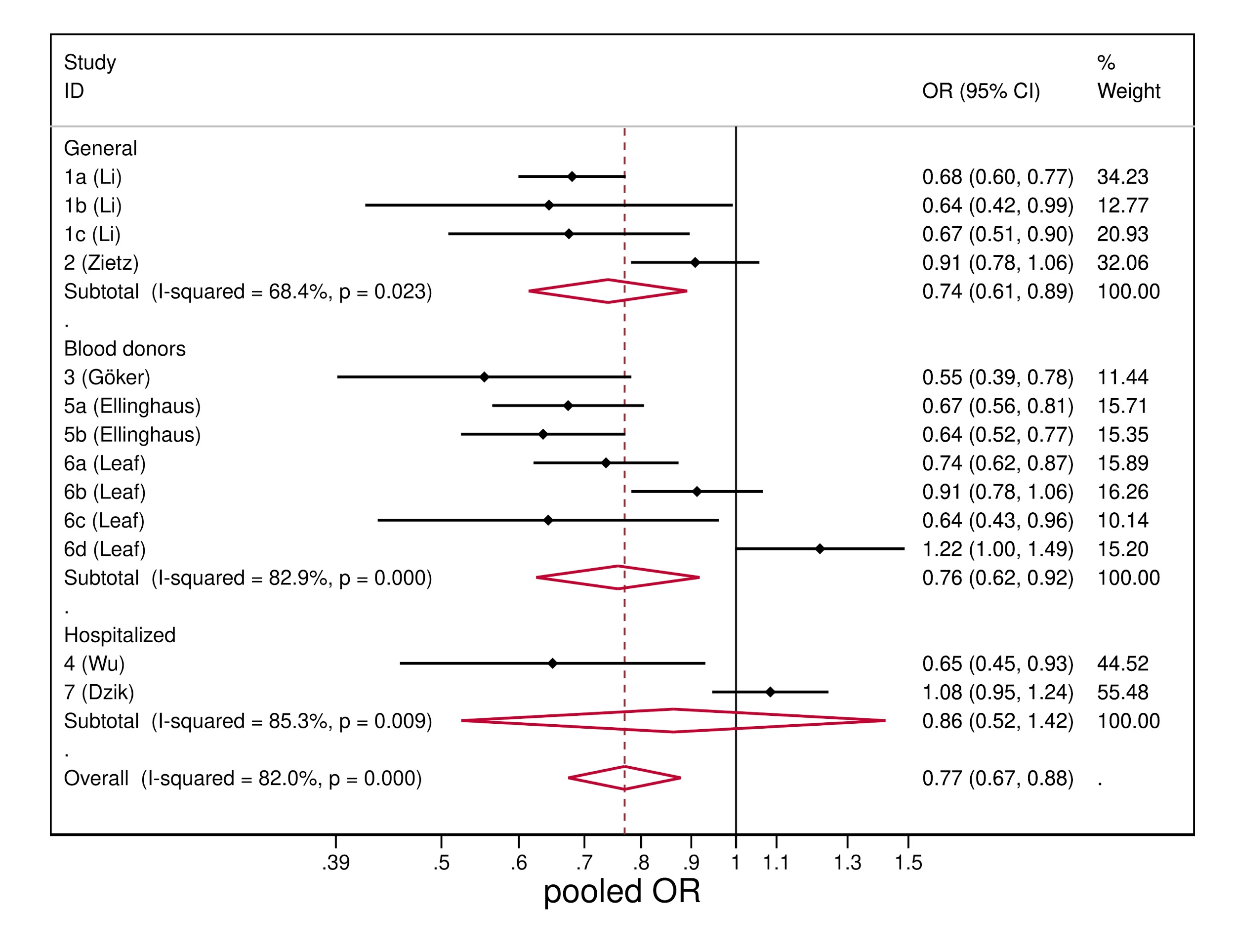


**Figure S4.** Forest plot from random effects analysis: OR of being blood group 0 in SARS-CoV-2+ group versus control group, by Country.


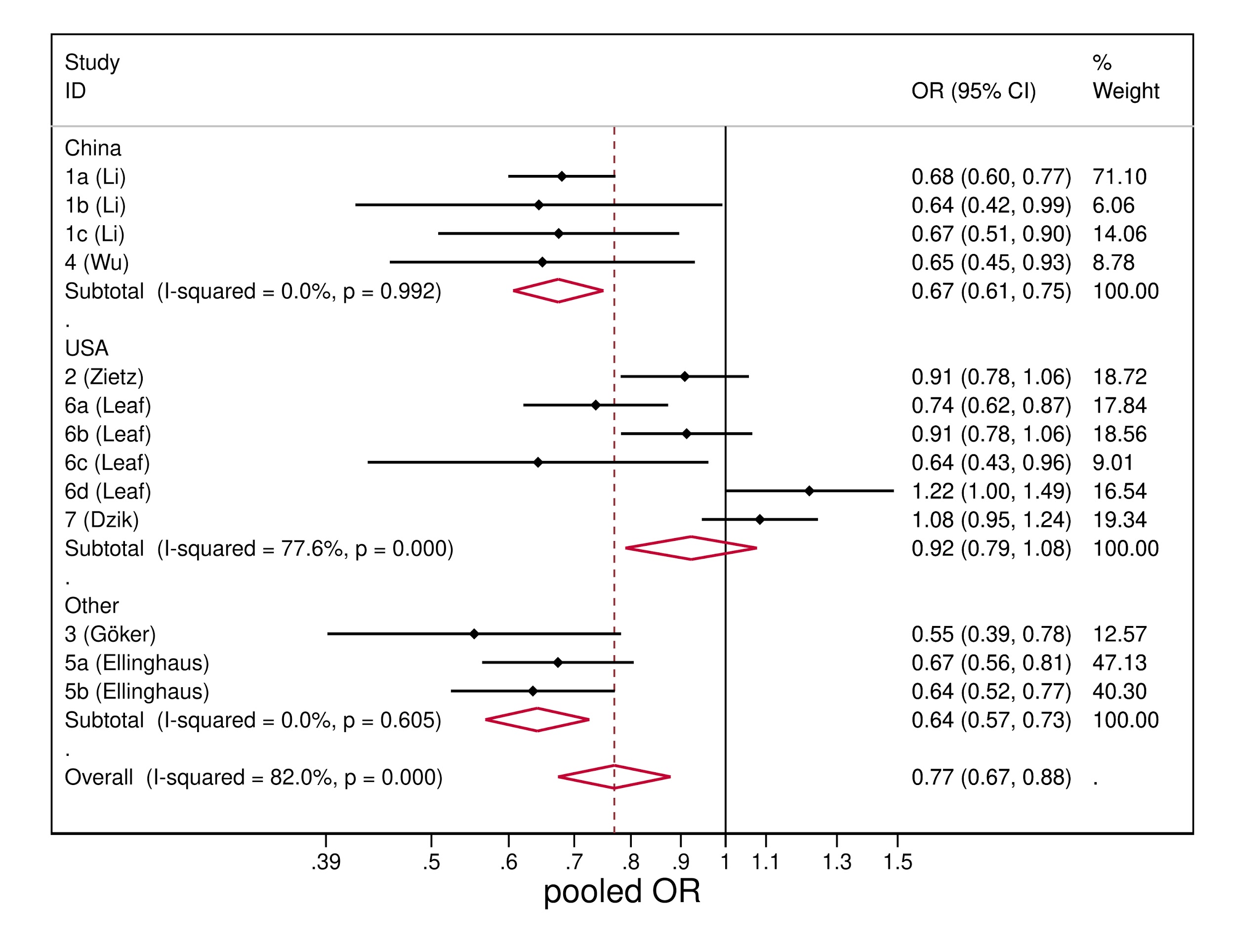

Supplement: S1 File — (DOCX) [file pone.0239508.s003.docx]
